# The Paradox of Prevention: How Prophylactic Surgery Accelerated Decline in Metastatic Cancer

**DOI:** 10.7759/cureus.107052

**Published:** 2026-04-14

**Authors:** Nicholas W Tyndall, Franchesca Farris-Cosme, Nicholas Phan, Andrew Ta, Dylan D Walker

**Affiliations:** 1 Internal Medicine, San Antonio Uniformed Services Health Education Consortium, San Antonio, USA; 2 Internal Medicine, University of Texas Health Science Center at San Antonio, San Antonio, USA

**Keywords:** frailty, metastatic prostate cancer, palliative, pathological femur fracture, prophylactic fixation

## Abstract

Metastatic castration-resistant prostate cancer (mCRPC) commonly involves bone metastases, with a high risk of pathological fractures that can lead to significant morbidity. Prophylactic fixation is often recommended based on scoring systems like Mirels. However, the decision to proceed with major surgery in elderly, frail patients with advanced disease poses complex challenges, as the physiological stress of surgery may outweigh its potential benefits.

We present the case of an 86-year-old man with end-stage mCRPC and high-risk bilateral proximal femoral lesions who underwent guideline-concordant prophylactic bilateral cephalomedullary nailing. Prior to surgery, a goals-of-care discussion was conducted in which the risks of postoperative complications and potential functional decline were reviewed; the patient demonstrated understanding and expressed a preference to proceed with surgery to reduce his pain and the risk of catastrophic fracture and preserve mobility. While the surgery successfully prevented fractures, his postoperative course was marked by severe anemia, transfusion reactions, hematuria, and a rapid functional decline. Within weeks, he became non-ambulatory and dependent on activities of daily living. Despite multidisciplinary management, he transitioned to home hospice within six weeks of surgery to prioritize comfort and quality of life. This case underscores the critical disconnect that can occur between technical surgical success and patient-centered outcomes in advanced cancer. While clinical guidelines and scoring tools like Mirels provide valuable direction, they do not account for patient frailty, life expectancy, or functional reserve. In this instance, the intervention may have inadvertently hastened the patient's decline, emphasizing the importance of early palliative care involvement and shared decision-making that incorporates discussions around prognosis, functional outcomes, and individual goals of care.

In patients with advanced malignancy and limited reserve, the physiological burden of prophylactic surgery may undermine their remaining quality of life. A nuanced, multidisciplinary approach is essential to align treatment plans with patient values and priorities.

## Introduction

Metastatic prostate cancer has a strong predilection for skeletal involvement, with bone metastases developing in more than 80% of men with advanced disease [1]. These lesions can lead to pathological fractures, which are fractures occurring in weakened bone due to cancer rather than trauma. These are often devastating events associated with significant pain, functional loss, and increased mortality [2]. Consequently, prophylactic surgical fixation is the standard of care for impending pathological fractures of the long bones, guided by validated scoring systems such as the Mirels score, a clinical tool used to estimate fracture risk in metastatic bone lesions [3,4]. However, the decision to proceed with major surgery in elderly, frail patients with a heavy disease burden and limited life expectancy presents a profound clinical challenge [5]. Frailty, reflecting reduced physiological reserve and increased vulnerability to stressors, is a key predictor of adverse postoperative outcomes in older adults and an increasingly important consideration in oncologic and surgical decision-making. Accordingly, management must balance the benefits of fracture prevention against the risks of postoperative complications and functional decline.

We present the case of an 86-year-old man with multiline refractory metastatic castration-resistant prostate cancer (mCRPC), a form of prostate cancer that continues to progress despite medical suppression of testosterone, and high-risk bilateral proximal femoral lesions. Following guideline-based recommendations, the patient underwent successful prophylactic cephalomedullary nailing, an intramedullary rod fixation technique placed within the femur to stabilize bone and prevent fracture. Despite preventing impending fractures, his postoperative course was marked by numerous complications and a catastrophic functional decline, leading to a complete loss of ambulation and independence. This case highlights the critical divergence that can occur between a technically successful surgical outcome and a patient's overall quality of life, illustrating how major physiological stressors can accelerate a patient's decline. By highlighting the role of frailty and limited functional reserve, this case underscores a critical gap in current practice, where guideline-driven decisions may not fully account for patient-specific vulnerability. It reinforces the importance of incorporating frailty, expected functional outcomes, and goals of care into shared decision-making for palliative surgical interventions.

## Case presentation

An 86-year-old man with a history of mCRPC, characterized by extensive skeletal metastases, refractory to multiple lines of treatment, presented for the definitive management of high-risk bilateral femoral metastases.

The patient had a complex treatment history: he was initially diagnosed with prostate adenocarcinoma in December 2014, with a Gleason score of 5+5=10 and a prostate-specific antigen (PSA) of 22 ng/mL (reference: <4 ng/mL). He was treated with androgen deprivation therapy (ADT) and external beam radiation therapy. In 2016, the patient developed sacral insufficiency fractures requiring surgical stabilization, after which ADT was discontinued. In 2020, surveillance imaging revealed disease progression with new osseous metastases. ADT was resumed, but was again discontinued due to intolerable side effects. From 2020 to 2024, he underwent multiple lines of systemic therapy, all of which were discontinued due to either adverse effects or continued disease progression.

**Figure 1:**
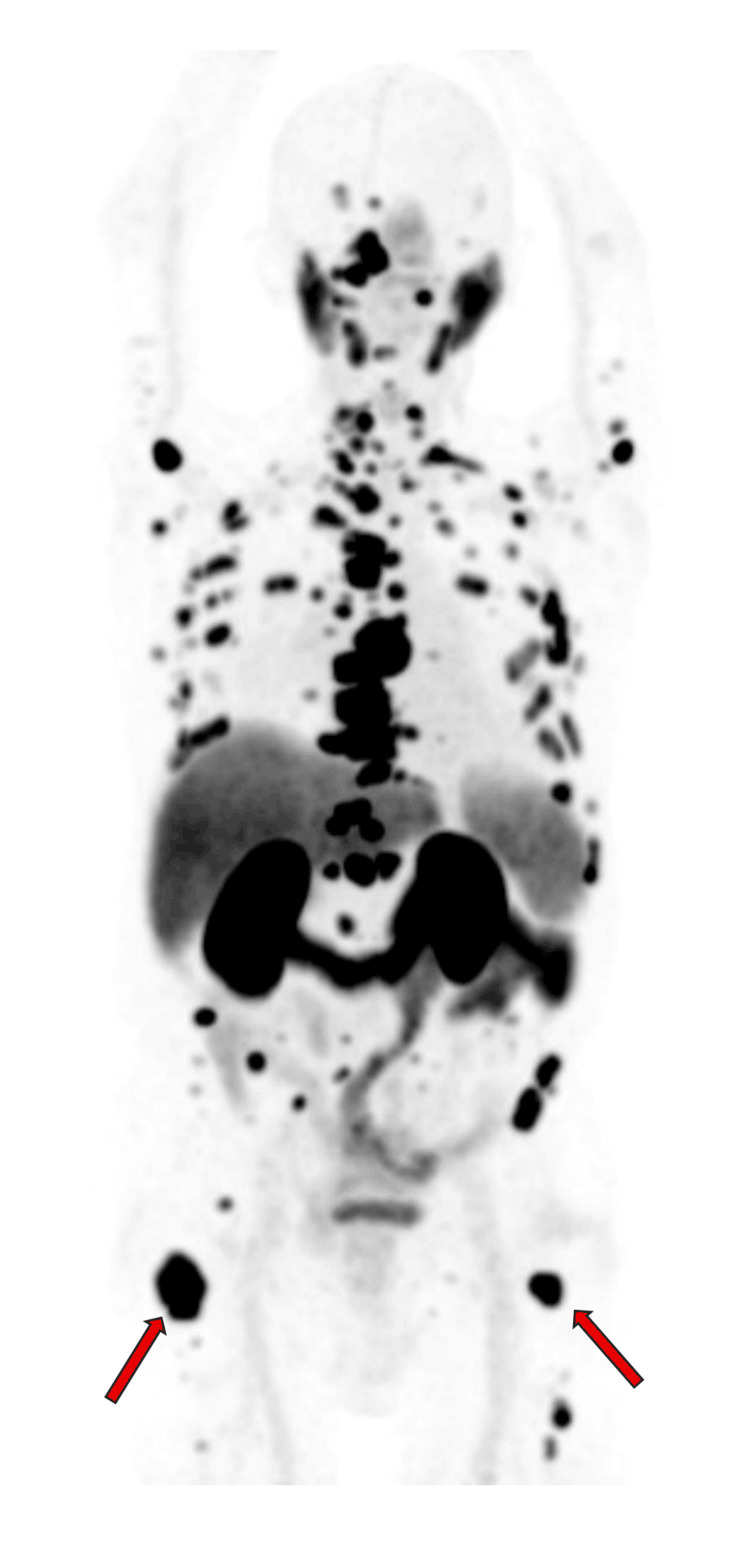
Whole-body PET/CT demonstrating extensive, diffuse osseous metastatic disease. Areas of increased radiotracer uptake (dark foci) are seen throughout the axial and appendicular skeleton, including the skull, cervical and thoracic spine, ribs, pelvis, and bilateral proximal femurs. Particularly intense uptake is noted in the bilateral proximal femurs (arrows), corresponding to high-risk lesions with a Mirels score of 9, indicating impending pathological fracture. Physiologic uptake is seen in the kidneys and bladder. These findings are consistent with widespread skeletal involvement from advanced metastatic prostate cancer PET: positron emission tomography; CT: computed tomography

In January 2025, positron emission tomography (PET)/computed tomography (CT) imaging (Figure 1) demonstrated extensive bilateral proximal femoral metastases with a Mirels score of 9 as seen in Table 1, indicating a high risk of impending pathological fracture [3].

**Table 1:** Mirels scoring for the patient's bilateral femoral lesions

Scoring criterion	Patient finding (bilateral)	Assigned score (1-3)
Site	Peritrochanteric (proximal femur)	3
Pain	Functional (pain with weight-bearing)	3
Lesion character	Blastic	1
Size	Involving 1/3 to 2/3 of the cortical diameter	2
Total Mirels score		9

A comprehensive goals-of-care discussion was conducted with the orthopedic surgery team, during which the risks and benefits of prophylactic fixation were reviewed, including the potential for postoperative complications given the patient's advanced age, frailty, and disease burden. The patient understood these risks and wanted to proceed with surgical intervention in order to prevent a potentially catastrophic fracture and preserve mobility for as long as possible. Given the urgent findings, the patient was referred to orthopedic surgery and underwent prophylactic bilateral cephalomedullary nailing. However, his postoperative course was complicated by significant issues. Shortly after surgery, he was found to have severe anemia with a hemoglobin level of 5.8 g/dL (reference range: ~13.5-17.5 g/dL), requiring multiple transfusions. The patient's hemoglobin trend and transfusion requirements throughout his hospitalization are summarized in Table 2. His hospital course was further complicated by a febrile non-hemolytic transfusion reaction and two episodes of presyncope, likely vasovagal in nature. Following the resolution of his anemia and presyncope symptoms, he was discharged home.

**Table 2:** Postoperative hemoglobin trends and transfusion requirements POD: postoperative day

POD	Hemoglobin (g/dL)	Reference	Transfusion required?
Day of surgery	8.0	13.5-17.5	No
POD1 AM	5.8	13.5-17.5	Yes, 2 units of packed red blood cells
POD1 PM	7.8	13.5-17.5	No
POD2 AM	7.2	13.5-17.5	No
POD2 PM	6.0	13.5-17.5	Yes, 1 unit of packed red blood cells
POD3 AM	7.6	13.5-17.5	No
POD4 AM	8.6	13.5-17.5	No
POD5 AM	7.7	13.5-17.5	No
POD6 AM	8.4	13.5-17.5	No
POD11 AM	7.5	13.5-17.5	No
POD12 AM	6.3	13.5-17.5	Yes, 1 unit of packed red blood cells
POD12 PM	7.6	13.5-17.5	No
POD13 AM	6.4	13.5-17.5	1 unit of packed red blood cells
POD13 PM	7.5	13.5-17.5	No
POD14 AM	6.2	13.5-17.5	1 unit of packed red blood cells
POD14 PM	7.9	13.5-17.5	No
POD15 AM	7.4	13.5-17.5	No
POD16 AM	6.7	13.5-17.5	1 unit of packed red blood cells
POD17 AM	8.0	13.5-17.5	No
POD18 AM	8.5	13.5-17.5	No
POD19 AM	8.6	13.5-17.5	No

Within one week, the patient returned to the hospital with failure to thrive, worsening leg weakness, and new hematuria. Hematuria progressed after the initiation of anticoagulation therapy for a partially occlusive internal jugular vein thrombus discovered in the emergency department before admission. Due to active bleeding, anticoagulation was discontinued. Urology was consulted, and continuous bladder irrigation was initiated, which proved ineffective. Radiation oncology was subsequently involved and provided palliative radiation therapy, which successfully controlled the bleeding. The patient was discharged home again with continued follow-up for further radiation to prevent bleeding.

Two weeks later, he was readmitted for further decline in functional status. Since the surgery, he had become non-ambulatory and reported profound weakness. He had also lost the ability to feed himself, now being able to complete no activities of daily living on his own. Given his continued deterioration and his long-term enrollment in hospice care, discussions were held with his hospice team. The patient and his wife opted to avoid skilled nursing facilities and agreed to continue care at home under hospice supervision with increased supportive services. He has since canceled all follow-up appointments, trying to enjoy his last days in hospice without going to the doctor. A full timeline since diagnosis can be found in Table 3.

**Table 3:** Clinical timeline of disease progression and postoperative course PET: positron emission tomography; CT: computed tomography

Time point	Event
2014	Initial diagnosis of prostate adenocarcinoma
2016	Sacral insufficiency fractures requiring surgical stabilization
2020-2024	Disease progression; multiple lines of systemic therapy attempted and discontinued
Jan 2025	PET/CT shows high-risk bilateral femoral lesions (Mirels score: 9)
Post-op days 1-5	Undergoes prophylactic bilateral cephalomedullary nailing. Develops severe anemia (hemoglobin level: 5.8 g/dL) requiring transfusions, a transfusion reaction, and presyncope
Post-op week 1	Readmitted for failure to thrive, worsening weakness, and new hematuria. Diagnosed with a partially occlusive internal jugular vein thrombus
Post-op weeks 1-2	Anticoagulation discontinued due to bleeding. Undergoes continuous bladder irrigation and subsequent palliative radiation therapy, which successfully controls the hematuria
Post-op week 4	Readmitted for further catastrophic decline in functional status; now non-ambulatory and unable to self-feed
Post-op week 6	After discussion with family and hospice team, the patient transitions to intensified home hospice care, ceasing all further clinical appointments

## Discussion

This case presents the challenging clinical course of an 86-year-old man with end-stage mCRPC who underwent prophylactic bilateral cephalomedullary nailing for impending pathological fractures. At the time of surgical evaluation, the patient had a poor baseline functional status, requiring assistance with ambulation and demonstrating features consistent with limited physiological reserve. While the surgical intervention was technically successful and followed established clinical guidelines, the patient's subsequent catastrophic functional decline and multiple complex medical complications underscore the profound challenges inherent in managing frail, elderly patients with advanced malignancies. This case highlights the importance of balancing guideline-directed intervention with a patient's limited physiological reserve, recognizing that major surgery in frail patients may contribute to functional decline, and the need for early, integrated multidisciplinary and palliative care in shared decision-making.

The decision to proceed with prophylactic fixation was well supported by the Mirels scoring system, a widely used tool to predict the risk of pathological fracture in long bones affected by metastatic disease [3]. This system assesses four criteria: lesion location, size, character (lytic, blastic, or mixed), and pain level. A score of 8 is considered the threshold for recommending prophylactic fixation, with scores of 9 or greater indicating a high risk where fracture is nearly inevitable without intervention [3,4]. Given that the patient's bilateral proximal femoral lesions both scored 9, the recommendation for surgical stabilization was consistent with the standard of care. Prophylactic surgery has been shown to result in better functional outcomes, shorter hospital stays, and lower complication rates than fixation after a fracture has occurred [2].

However, the benefits of fracture prevention must be weighed against the substantial risks of major surgery in frail elderly patients with a heavy burden of disease. Frailty, which can be defined by factors such as unintentional weight loss, weakness, and low physical activity, is a marker of decreased physiological reserve and an independent predictor of adverse postoperative outcomes in older surgical patients, including higher rates of complications and discharge to skilled nursing facilities [5]. In this case, the patient exhibited clinical features consistent with frailty; however, a formal preoperative assessment using a validated tool (e.g., Clinical Frailty Scale, FRAIL scale, or Fried Frailty Phenotype) was not performed. This represents a limitation, as standardized measures may have provided a more objective assessment of surgical risk and functional reserve. 

The patient's postoperative course was marked by severe anemia requiring transfusion, a transfusion reaction, and presyncope, reflecting this underlying vulnerability. Evidence from prospective studies of older adults undergoing hip fracture surgery demonstrates a notable early decline in functional status and quality of life, with mean Barthel Index scores, a measure of independence in activities of daily living such as feeding, mobility, and self-care, decreasing from approximately 88 pre-fracture to 73 at three months, followed by only partial recovery to around 75 at one year, indicating that many patients do not return to their preoperative baseline [6]. The readmissions for failure to thrive and hematuria further illustrate how a single major intervention can trigger a spiral of decline in a patient with multisystemic disease. This case supports the routine incorporation of validated frailty assessments into preoperative evaluation, particularly in elderly patients with advanced malignancy, to better inform risk stratification and shared decision-making.

This patient's rapid deterioration highlights a crucial distinction between achieving a surgical goal and achieving a patient-centered goal. The operation successfully prevented bilateral femoral fractures, but at the cost of the patient's remaining independence and quality of life. Within weeks of the procedure, he transitioned from being ambulatory with assistance to being non-ambulatory, complete dependence for feeding, and requiring total care. This outcome raises difficult questions about the true benefit of the intervention in the holistic context of the patient's life. While predicting such a precipitous decline is challenging, it underscores the need for preoperative discussions that frame potential outcomes not only in terms of fracture prevention but also in terms of the realistic risk of global functional loss [7]. For patients with limited life expectancy and borderline performance status, maintaining quality of life and preserving independence may be a higher priority than preventing a future event, even a devastating one.

The management of this patient's multiple complications necessitated a robust multidisciplinary approach involving orthopedic surgery, medical oncology, urology, radiation oncology, and hospice care. His course was complicated by hematuria, a known consequence of advanced prostate cancer with direct bladder or ureteral invasion [8]. While continuous bladder irrigation was attempted, the definitive management required palliative radiation therapy, a well-established and highly effective modality for controlling tumor-related hemorrhage from various sites, including the pelvis [9]. The successful hemostasis achieved with radiation underscores its vital role in the palliative setting. The ultimate transition to intensified hospice services reflects the recognition by the patient, family, and clinical team that the patient's overall trajectory was one of decline and that the goals of care had shifted firmly to comfort and quality of life. The American Society of Clinical Oncology (ASCO) strongly recommends the early integration of palliative care for patients with advanced cancer to facilitate these complex discussions and ensure that treatment decisions remain aligned with patient values as their clinical status evolves [10].

## Conclusions

This case serves as a powerful illustration of the complex decision-making required for patients with advanced cancer and impending pathological fractures. While the Mirels score provides a clear indication for surgical intervention, it does not account for patient frailty or the potential for the intervention itself to precipitate a global decline. The prevention of a pathological fracture is a laudable and important goal, but for some patients, the physiological cost of major surgery may outweigh the benefit. This case argues for a nuanced approach to shared decision-making that explicitly discusses the risk of functional loss and ensures that interventions, even when technically indicated, align with the patient's most important goals, whether it is survival, fracture prevention, or the preservation of quality of life and independence. In practice, this may be supported by routinely incorporating frailty assessments into preoperative evaluation, engaging multidisciplinary teams in cases where the benefit of surgery is less certain, and involving palliative care early in the decision-making process for patients with advanced malignancy undergoing consideration of prophylactic procedures. These steps may help better contextualize surgical risk within the broader framework of prognosis, functional reserve, and patient-centered priorities.
